# Azelastine hydrochloride and budesonide (nasal sprays): effectiveness combination therapy monitored by acoustic rhinometry and clinical symptom score in the treatment of allergic rhinitis

**DOI:** 10.1186/2045-7022-3-S2-P40

**Published:** 2013-07-16

**Authors:** Natalia Zanellato Fabbri, Ricardo de Lima Zollner, Eduardo Abib Junior

**Affiliations:** 1 University of Campinas, Campinas, Brazil; 2 University of Campinas, Department of Internal Medicine – Faculty of Medica, Campinas, Brazil

## Background

Guidelines therapy for allergic rhinitis (AR) recommends new-generation H1-antihistamines and intranasal corticosteroids as main treatment. The objective for this study was to evaluate the effects of intranasal therapy with azelastine hydrochloride and budesonide (isolated and combined) using nasal provocation test (NPT) and acoustic rhinometry in patients with AR.

## Methods

The study population consisted of 28 patients (9 female and 19 males, aged between 18-32 years) with diagnostic of persistent AR (ARIA consensus). This was a randomized, crossover study. Subjects were randomly assigned to receive either azelastine hydrochloride (140 mcg/nostril) or budesonide (64mcg/nostril) or both drugs. All patients received the three treatments using nasal spray twice daily, each period of treatment lasted 30 days and washout period was 7 days. Subjects were submitted to nasal provocation test (NPT) with histamine before and after each period of treatment. Nasal responsiveness to histamine was monitored based on subjective (symptom score) and objective parameters (acoustic rhinometry) to compare the treatments. After acoustic rhinometry measure (baseline) histamine was instilled in nasal cavity (0.5mg/mL/nostril) via nasal spray. Minimal cross-area (MCA2) was measured by acoustic rhinometry at times 1; 4; 8; and 12 minutes after NPT for each histamine concentration (0.5; 1; 2; 4 and 6 mg/mL) up to positive response occurs (nasal obstruction). The criteria for a positive response were histamine dose and rhinometric measure time causing at least 20% fall in MCA2 (NPT20). NPT was stopped when a positive response occurred. Statistical significance was assessed by paired t-test.

## Results

Baseline MCA2 decreases significantly after azelastine therapy (p=0.01) and did not change with budesonide. Combination therapy demonstrated significantly increasing in baseline MCA2, namely improvement of nasal patency (p=0.005). MCA2 after NPT increased with budesonide (p=0.02) and combined therapy (p=0.002), demonstrating the protective effect of therapy. There was a decrease in MCA2 NPT20 after azelastine hydrochloride therapy (p=0.02). Score symptoms during NPT decreases significantly after all treatments (azelastine p=0.04, budesonide and combined drugs p<0.0001).

## Conclusion

Azelastine hydrochloride therapy combined with budesonide might provide more therapeutic benefits than isolated drugs in nasal patency and symptoms score in patients with AR.

